# Supplementary material for: Autism sensory dysfunction in an evolutionarily conserved system
Source: Proc Biol Sci. 2018 Dec 12;285(1893):20182255. doi: 10.1098/rspb.2018.2255 (PMC6304042; doi:10.1098/rspb.2018.2255)
Supplement: ESM [file rspb20182255supp1.zip › Figshare_ProcRSocB/README.docx]

This document describes the purpose and use of the main variables and functions contained within this repository. The workflows for each data set (ASD/NT adults, AQ adults, ASD/NT children and fruit flies) are described separately. Modelling of the CRFs is described at the end.

**Workflow ASD/neurotypical adults**

**analyseindividual_adults.m**

This script is for reference on how the **Sxx_processed.mat** files were created from raw EEG files for all adults (both ASD and AQ data sets) in the study

**makegroupCRFs_ASDadults.m**

This script takes the **Sxx_processed.mat** and combines them into a group file **ASDadults_CRFs.mat**

**plotgroupCRFs_ASDadult.R**

R script that plots the adult ASD and neurotypical control data as figures for the paper

**Sxx_processed.mat files**

*Many variables in these files have a contrast condition dimension of size 14 (1-7 target contrast conditions only; 8-14 target contrasts + 32% contrast orthogonal mask at 5Hz)*

alphapower – contains averaged alpha power over 8-12Hz. Dimensions: electrode x contrast conditions x repetitions

channelmappings – variable used to map the row of data variables to ANTneuro scalp electrode montage for headplots

evokedresp – full fourier transform taken over each 10s trials (using fft). Dimensions: electrodes x contrast conditions x repetitions x fourier spectrum

imF – intermodulation term (5+7Hz = 12Hz)

imF2 – second intermodulation term (24Hz)

maskF – amplitude at the mask frequency (5Hz)

maskF2 – second harmonic at mask frequency (10Hz)

SNRim, SNRmask, SNRtarget – signal-to-noise ratios

targetF - amplitude at the target frequency (7Hz)

targetF2 – second harmonic at target frequency (14Hz)

**ASDadults_CRFs.mat file**

Contains processed and averaged CRFs for ASD (all variables start with asd…) and neurotypical (all variables start with typ…) groups. This is what the R plotting script uses to produce plots.

**Workflow AQ adults**

**Sxx_processed.mat files**

Same as for the ASD adults described above

**analyseindividual_adults.m**

This script is for reference on how the **Sxx_processed.mat** files were created from raw EEG files for all adults (both ASD and AQ data sets) in the study

**plotgroupCRFs_AQadult.R**

R script that plots the CRFs from the adult AQ dataset as two mean functions (low and high AQ)

**plot_AQ_regression.R**

R script that plots a scatterplot (Figure 4 in the paper) to show the regression analysis of the adult AQ dataset

**AQadults_CRFs.mat files**

Contains aggregated data from the 100 participants from the neurotypical AQ dataset. Important variables:

AQ – AQ scores on the short AQ questionnaire (max score 28)

alltargetF1, alltarget2F1 – first and second harmonic responses for each subject, contrast condition (1-7 target contrast conditions only; 8-14 target contrasts + 32% contrast orthogonal mask at 5Hz) and repetition (8 in each condition).

mean_targF1, mean_targ2F1 – mean responses for each subject (averaged coherently over repetitions) for the unmasked contrast conditions. These were calculated from alltargetF1 and alltarget2F1

levelsC, levelsdB – target contrast levels used in Michaelson contrast and in dB

**Workflow ASD children**

**Raw_cXXX_tXXX.mat**

In the Children_data folder. Contains raw EEG data from each condition (c) and repetition (t).

RawTrial – dimensions: time (420Hz sampling rate, 5040 samples total) x electrodes (130)

**makegroupCRFs_children.m**

Analyses matlab files in the ‘Children_data’ folder and aggregates data into contrast response functions averaged over repetitions for each individual participant. Produces file ASDchildren_CRFs.mat

**children_CRFs.mat**

Variables contained in this file are separate for the ASD and neurotypical children. Variables containing full CRFs (dimension length = 12) contain all contrast level bins throughout the trials. The first and two last bins of each trial were not used in the analysis or figures.

ASDcondratio / TYPcondratio – mean 1F/2F ratio (over contrast conditions in indexes 6,7,8,9,10) for each participant

ASDcondSE / TYPcondSE – between subject standard error for the mean 1F/2F ratio

ASDfreqRatio / TYPfreqRatio – 1F/2F ratios for each participant and each contrast bin

ASDgood1F / TYPgood1F - amplitude at the target frequency for each participant and contrast bin after rejecting bad epochs

ASDgood2F / TYPgood2F - amplitude at the second harmonic frequency for each participant and contrast bin after rejecting bad epochs

ASDmeanratio / TYPmeanratio – mean 1F/2F ratio (over contrast conditions in indexes 6,7,8,9,10; and over participants)

ASDweight1F / TYPweight1F / ASDweight2F / TYPweight2F – weighted means over participants to account for bad bins

seASDfreqRatio / seTYPfreqRatio – standard error f

seASDweight1F / seTYPweight1F / seASDweight2F / seTYPweight2F

–standard errors over participants to account for bad bins when calculating weighted means

subWeight_asd / subWeight_typ – number of ‘good’ bins used to weight the means (out of 10 repetitions)

**plotgroupCRFs_children.R**

R script that creates figures used in the paper for the ASD/NT childrens data. Uses the children_CRFs.mat file.

**Workflow *Drosophila***

Unprocessed ERG data for the fruit flies are not provided here as they were recorded as part of a large sample of fruit flies, and extracted from a large database (used for other experimental purposes) with a total of 328 experiments (656 fruit flies; around 2000 .mat files).

**analyseindividual_fruitflies.m**

This script is provided for reference for how the data was extracted from the database and processed. This pipeline is very similar to those used for human data.

**fruitflies_CRFs.mat**

This file contains data for the six fruit fly groups, in order: 3-day-old NHE^KG08307^, NHE^KG08307^/Df(2LBSC187), wildtype (CS/iso); 14-day-old NHE^KG08307^, NHE^KG08307^/Df(2LBSC187), wildtype (CS/iso).

allflies1f – target frequency (12Hz) response for the six genotypes (as described above). Dimensions: 6 genotypes; 12 flies per genotype, 14 contrast conditions (1-7 target only, 8-14 target + mask at 15Hz)

allflies2f – second harmonic response (24Hz) for the six genotypes (as described above). Dimensions: 6 genotypes; 12 flies per genotype, 14 contrast conditions (1-7 target only, 8-14 target + mask at 15Hz)

condratio – 1f/2f ratios for all contrast conditions, flies and genotypes

meancondratio – mean 1f/2f ratio for the six fruit fly genotypes, calculated over contrast conditions that had responses above the noise floor. Averaged over flies within genotypes

meanmask1f, meanmask2f – mask frequency responses (15Hz and 20Hz)

meanratio – mean 1f/2f ratios for each contrast condition, averaged over fruit flies within the genotypes.

meantarget1f, meantarget2f – first and second target responses averaged over fruit flies within the genotypes

SEcondratio – standard error over flies for the mean 1f/2f ratio

SEmask1f, SEmask2f – standard error for the mean mask responses, calculated over flies

SEratio – standard error for the 1f/2f ratio within each contrast condition, calculated over flies

SEtarget1f, SEtarget2f - standard error for the mean target responses, calculated over flies

**Modelling**

makemodelfits.m – fits a normalization model to each group within each data set and outputs the curves and parameters. Bootstraps the fits to obtain 95% confidence intervals.
